# The results of external quality assessment programme on urine leukocyte and erythrocyte counting in Poland

**DOI:** 10.11613/BM.2020.020707

**Published:** 2020-06-15

**Authors:** Agnieszka Ćwiklińska, Robert Kowalski, Barbara Kortas-Stempak, Agnieszka Kuchta, Aleksandra Fijałkowska, Gabriela Bednarczuk, Maciej Jankowski

**Affiliations:** 1 Department of Clinical Chemistry, Medical University of Gdańsk, Gdańsk, Poland; 2 Hospital Pharmacy, University Clinical Centre, Medical University of Gdańsk, Gdańsk, Poland; 3 SOWA-med Sp. z o.o., Gdańsk, Poland

**Keywords:** erythrocytes, leukocytes, quality control, standardisation, urine

## Abstract

**Introduction:**

Urine particle analysis is an important diagnostic tool. The aim of this study was to evaluate the quality of urine leukocyte (WBC) and erythrocyte (RBC) counting results obtained with manual and automated methods in Polish laboratories participating in the international external quality assessment (EQA) programme.

**Materials and methods:**

1400 WBC and RBC counting results were obtained from 183 laboratories in EQA surveys organised by Labquality (Helsinki, Finland) from 2017 to 2019. The between-laboratory coefficient of variation (CV), the percentage difference between the laboratories' results and target values (Q-score (%)), as well as modified Youden plots were analysed.

**Results:**

For automated method groups, the medians of inter-laboratory CVs varied from 14% to 33% for WBC counting and from 10% to 39% for RBC counting. For manual method groups, the medians of CV varied from 53% to 71% (WBC) and from 55% to 70% (RBC), and they were significantly higher, in comparison to CVs for most automated method groups (P < 0.001). The highest percentage of results outside the target limits (36%) and the highest range of Q-score (%) (from - 93% to 706%) were observed for laboratories which participated in the surveys for the first or second time. The percentage of deviating results and the ranges of Q-score decreased with an increased frequency of laboratories’ participation in the surveys.

**Conclusions:**

The quality of manual methods of urine WBC and RBC counting is unsatisfactory. There is an urgent need to take actions to improve laboratories’ performance and to increase harmonisation of the results.

## Introduction

Urine particle analysis is an important diagnostic tool for renal and urinary tract diseases (*1*). To enable the achievement of a proper clinical outcome of the analysis, high quality examination results must be provided, which is dependent on good performance of the applied method and the good skills of the laboratory staff in the recognition of urine particles (*2*-*4*).

Manual microscopic and automated methods are used for urine particle analysis. Automated analysers have better accuracy and precision than manual methods (*5*). Moreover, their application enables the reduction of time, labour and cost of analysis (*6*). However, they have limitations in the recognition of urine elements, especially in highly pathologic samples (*7*, *8*). For that reason, microscopic methods are still considered as the ‘gold standard’ (*1*, *2*).

Different microscopic methods are distinguished, namely, urine sediment counting under a coverslip, urine sediment counting in a chamber, and direct chamber counting. The most popular is the coverslip method applied in routine practice due to its easy performance and long tradition of using in laboratories (*2*, *9*). Many laboratories still apply the non-standardised coverslip method with decanting supernatant after centrifugation and inserting the drop of sediment on the slide. Thus, the low precision and wide uncertainty of results obtained with the coverslip method is observed (*2*, *10*). Chamber use standardises the volume of sample analysed under the microscope and analysis of uncentrifuged urine enables to avoid errors related with the centrifugation step. Thus, the direct chamber method is useful as a comparison method in the evaluation of automated analysers. However, the analysis of uncentrifuged samples in routine practice can result in a lower sensitivity of urine analysis since a significantly lower number of urine elements is present in the analysed sample volume under the microscope (*2*).

Moreover, different protocols for manual methods can be applied in laboratories, varying in type of analysed specimen (stained or unstained), type of microscope used (bright-field or contrast-phase), the manner of expression of the results (*per* high power field (HPF) or in a volume unit). If a centrifuged sample is analysed, laboratories can also apply a different force and time of centrifugation, as well as the manner (decanting, using a pipette) and degree of urine concentration (*2*, *10*-*12*). Thus, despite the availability of recommendations/guidelines for urine particle analysis, the standardisation of urine particle analysis procedures is poor (*2*, *10*-*13*). This can be related to the fact that there are many different urine particle methods, but there is no reference method/procedure. Furthermore, there is no one worldwide standard for urine particle counting analysis, and various guidelines suggest different protocols (*2*, *12*). Moreover, some laboratories may not afford the additional costs that can be required for the implementation of the standardised procedures or they may not be aware of the necessity of implementing such procedures (*10*, *12*).

The lack of procedure standardisation leads to poor harmonisation of the obtained results that we observed in 2010 when assessing the quality of the urine leukocyte (WBC) and erythrocyte (RBC) counting results obtained by Polish laboratories in an external quality assessment (EQA) scheme provided by Labquality (Helsinki, Finland). We found that there was a high dispersion of the results, especially in the group of manual methods, when a non-standardised (non-quantitative) procedure of sediment preparation was applied (*10*). For the last ten years, educational activities such as courses for laboratory staff and lecturers at national conferences, focusing on the role and ways of urine particle analysis standardisation have been provided in Poland (*14*). Polish laboratories participating in EQA surveys have also received detailed instructions for standardised manual urine sediment analysis with the control specimen. However, these activities had no official recommendation status, and thereby did not result in a mandatory implementation of the standardised procedures in laboratories.

Under the auspices of the Polish Society of Laboratory Diagnostics, guidelines for urine particle analysis, which are intended to be mandatory in Poland, have been recently prepared (*15*). They are based on the European Federation of Laboratory Medicine (EFLM) and Clinical and Laboratory Standards Institute (CLSI) guidelines, and cover all aspects of urine particle analysis including patient’s preparation, collection of the samples, methods of urine particle analysis, presentation of the results, and quality control (*2*, *13*). According to the Polish guidelines, the coverslip method is not recommended for using in routine practice, all laboratory procedures should be performed quantitatively, and WBC and RBC results should be expressed as the number of particles x10^6^/L (*15*).

The aim of this study was to evaluate the present quality of the WBC and RBC counting results obtained by Polish laboratories participating in the EQA programme organised by Labquality (Helsinki, Finland), after several years of providing educational activities in Poland, and preceding the implementation of national guidelines.

## Materials and methods

### Materials

We analysed 1400 results of urine WBC and RBC counting obtained from 183 different Polish laboratories in 10 consecutive ‘Urine strip test and particle count’ surveys, organised by Labquality (Helsinki, Finland) from March 2017 to July 2019.

The ‘Urine strip test and particle count’ scheme is accredited according to EN ISO/IEC 17043 (PT02/FINAS). It is organised four times a year. The control sample is a liquid or lyophilised preparation of human urine with added chemicals, stabilised human RBC and latex or organic particles to simulate WBC, and is dedicated to quality assessment of urine analysis, namely strip tests (blood, glucose, ketones, nitrite, pH, protein, WBC), estimation of density (specific gravity, osmolality), creatinine, and urine particle counting. The assessment of performance of urine particle counting method applied in the laboratory is based only on WBC and RBC results, since there is no possibility to prepare a stable urine control sample containing other urine particles such as epithelial cells or casts.

The results of WBC and RBC counting are expressed as the number of particles x10^6^/L. The laboratories are grouped by method in the case of using manual protocols or by analyser, in the case of using the automated method.

The results of Polish laboratories were classified in 13 method groups: 3 manual (Standardised sediment examination under a coverslip (Coverslip method), Chamber counting from sediment, Direct chamber counting), 9 automated (Analysers A-I), and the Other method group. To identify and categorise the particles in urine samples, 6 automated analysers use microscopic image-based technology, 3 – fluorescent flow cytometry. The distribution of results according to the applied method is presented in Table 1.

**Table 1:** Distribution of Polish laboratories’ results according to the method group, in ‘Urine strip test and particle count’ 1/2017-2/2019 surveys

**Method groups**	**Percentage of results**
Manual	63
Standardised sediment examination under a coverslip (Coverslip method)	39
Chamber counting from sediment	15
Direct chamber counting	9
Automated	35
Image-based technology	30
(Analysers A, B, C, D, H, I)	(6, 3, 5, 12, 1, 3)
Fluorescent flow cytometry	5
(Analysers E, F, G)	(1, 3, 1)
Other method	2

### Methods

In our study the results within 7 method groups have been analysed: 3 manual (Coverslip method, Chamber counting from sediment, Direct chamber counting) and 4 automated (Analyser A, Analyser B, Analyser C, Analyser D). The results of Analyser E-I and Other method groups were not statistically evaluated, since there was a small number of results obtained from Polish participants (< 3 per survey; Analyser E-H, Other method) or the method group was represented only in 2 surveys (Analyser I). The names of the evaluated analysers have been blinded in our study since the dispersion of the results for automated methods observed in EQA could be related not only to the analyser performance, but also to individual laboratory performance, and the use of EQA data to suggest the superiority or inferiority of particular urine analysers may be deceptive and misleading.

Thirteen extreme outliers (0.9% of the results), which differed from the target values more than 20-fold, were excluded from the analysis. These results were obtained in 4 laboratories, which reported 6, 4, 2 and 1 extreme outliers, respectively. Nine outliers were obtained in the Coverslip method group, and 4 in the Chamber counting from sediment group. Also, 16 results from a laboratory which reported results from test strips instead of particle counting, were excluded from the analysis.

With the 4/2017 and 1/2018 surveys, the questionnaire regarding the methodological aspects of urine particle counting was sent to laboratories. The questionnaire covered the particle counting method and, in the case of using the manual method, the details of the examination procedure such as urine volume, centrifugation force and time, sediment volume, the coverslip and microscopic view field dimensions or chamber type (*10*). 39% (23/59) and 24% (15/62) participants of the 4/2017 and 1/2018 survey, respectively, replied to the questionnaire. The overall response rate for laboratories that applied manual methods was 35% (28/79).

### Statistical analysis

For WBC and RBC counts in individual surveys for each method group the between-laboratory coefficient of variation (CV) was calculated. The normality of CV distribution was assessed using the Shapiro-Wilk test. Since in some method groups the data did not have normal distribution, the between-laboratory CVs for method groups are presented as medians and interquartile ranges (25^th^-75^th^ percentile), and they were compared using the Kruskal-Wallis test with Dunn’s post-hoc test.

For the result of each laboratory, the percentage (%) of difference between the reported result and method-specific target value was calculated as follows:

Q-score (%) = [(laboratory result – target value) / target value] x 100 (*16*).

The target value was established according to Labquality and it was a mean value calculated from the results obtained from participants from all countries in the method group that fall within the calculated limit: the median value of the uncorrected results ± 3 x uncorrected standard deviation. The target limit was established according to Labquality as a method-specific target value ± 50%. The results outside the target limit have been considered incorrect (unacceptable), and the percentage of such results has been calculated for the analysed method groups.

For each survey, a modified Youden plot was prepared, in which each point corresponded to the WBC and RBC counts obtained in one laboratory. P < 0.05 was established to be statistically significant. The analyses were performed using the Graph-Pad Prism 4.0 software (GraphPad Software Inc. San Diego, CA).

## Results

**Figure 1:**
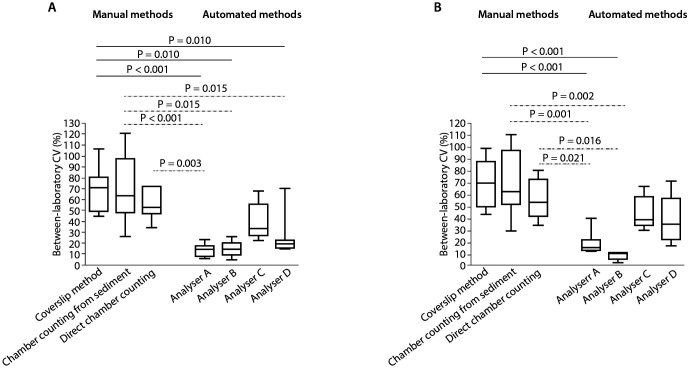
Between-laboratory coefficients of variation (CV) of Polish laboratories results according to the method in ‘Urine strip test and particle count’ 1/2017-2/2019 surveys. A. WBC counting, B. RBC counting. Data are presented as median, 25^th-^75^th^ percentiles, and minimum and maximum.

The medians of between-laboratory CV obtained for groups differed statistically significantly (P < 0.001). For WBC counting, the medians of CV for manual methods varied from 53% (47-72%; 25^th^-75^th^ percentiles) for the direct chamber counting method to 71% (49-81%) for the coverslip method, and were significantly higher in comparison to medians of CV for most automated method groups, which varied from 14% (8%-18%) for Analyser A to 33% (27%-56%) for Analyser C (Figure 1). For RBC counting, the medians of CV for manual methods varied from 55% (43%-74%) for direct chamber counting to 70% (51%-89%) for the coverslip method, and for automated method groups they varied from 10% (6%-11%) for Analyser B to 39% (35%-58%) for Analyser C (Figure 1).

33% of the results of manual methods and 12% of the results of automated methods were outside target limits. For two automated method groups (Analyser A and B) all results were within the target limits.

**Figure 2:**
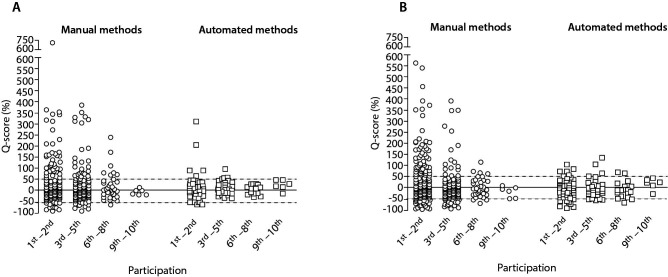
The percentage differences between the results of Polish laboratories and target values (Q-score (%)) according to the frequency of participation in ‘Urine strip test and particle count’ 1/2017-2/2019 surveys. A. WBC counting, B. RBC counting. The dotted line indicates the target limit (target value ± 50%).

For both manual and automated methods, the highest percentage of results outside the target limits (36% and 15%, respectively) and the highest ranges of Q-score (%) (from -93% to 706% and from -88% to 310%, respectively) were observed for laboratories which participated in the analysed EQA surveys for the first or second time (Figure 2). The percentage of unacceptable results and the ranges of Q-score (%) decreased along with an increased frequency of participation in the surveys, and for laboratories which participated in surveys for the ninth or tenth time, all results were acceptable and within the target limits (Figure 2).

**Figure 3:**
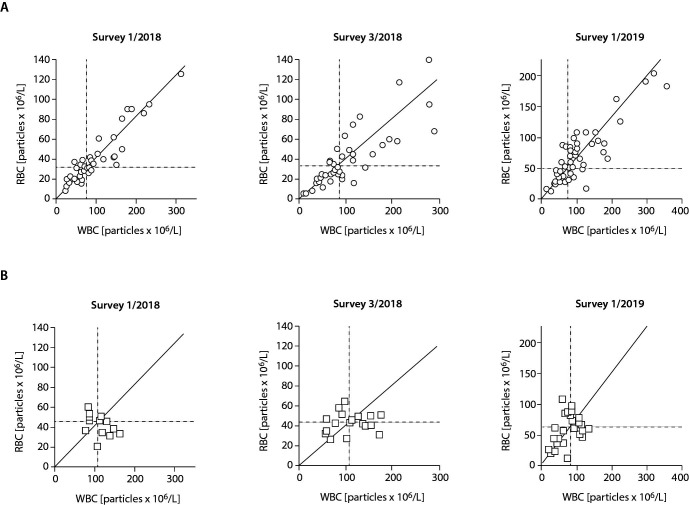
An exemplary modified Youden plots of the Polish laboratories results obtained in ‘Urine strip test and particle count’ 1/2017-2/2019 surveys. A. Manual methods, B. Automated systems. The dotted line indicates the median values of obtained results. The intersection of the two median lines indicates the Manhattan median. The solid line is a 45-degree line drawn through the Manhattan median.

For manual methods, the modified Youden plots showed many points that were far from median values, but near a 45-degree line. For automated methods, most points were near the median values and they did not run along a 45-degree line (Figure 3).

## Discussion

In our study based on EQA results we established that the quality of manual methods of urine WBC and RBC counting was unsatisfactory, and the dispersion of results obtained by laboratories using automated methods was significantly lower in comparison to those using manual methods. We also found that the systematic errors related to the laboratories’ procedures was a source of the high dispersion of results for manual methods, and the dispersion of results was related to the frequency of participation in EQA surveys.

In many previous studies automated systems have been shown to provide better precision than manual methods (*1*, *5*, *17*). In our study, the dispersion of results obtained for two analysers was comparable to those obtained in studies assessing the performance of automated methods, whereas for two others it was higher (*18*, *19*). The observed higher dispersion of results in our study could be affected by the performance of individual laboratories that use a particular analyser type, for instance different protocols of verification of analyser results could have been applied (*6*, *20*, *21*). Thus, it can be assumed that the dispersion of EQA results for automated method groups could better reflect the real-life quality of the results of automated methods, since it is related to both, analyser performance and laboratory performance.

For manual methods, the dispersion of results was comparable to those observed in our previous study performed in 2007-2010 (*10*). The failure in improving the overall quality of the results of manual methods during the last decade could be due to the widespread use of the traditional, non-standardised coverslip method in Polish laboratories, the limited number of laboratories that could participate in organised courses and lectures, that had only educational nature and did not result in a mandatory implementation of standardised procedures. This could be also due to the fact that standardised procedures of manual urine particle analysis are more time-consuming and labour-intensive than traditional, non-standardised procedures and their implementation would require an increase in financial outlays and laboratory staff involved in the analysis. However, we have observed that in comparison to EQA surveys performed in 2007-2010, the number of results per survey obtained in 2017-2019 has almost doubled, which may indicate a growing awareness of the role of participation in EQA surveys in improving the quality of urine particle counting among Polish laboratories (*10*). There was also a significant shift toward automated methods – the number of participants using analysers increased almost 3-fold, while the number of participants using the coverslip method decreased (*10*).

We have also previously found, that the laboratories using a manual standardised qualitative procedure exhibited lower dispersion of results in comparison to those using a non-standardised procedure (*10*). In the present analysis, we were not able to divide laboratories according to the applied procedure, but the modified Youden plots for manual methods showed again that the high dispersion of results was mainly caused by a laboratory systematic error, i.e. the applied procedure and/or calculation of results (*10*, *16*). Moreover, we found that the dispersion of results was related to the frequency of participation in surveys. This could indicate that the laboratories which obtained unsatisfactory results in EQA took action aiming at improving the quality of obtained results. Indeed, some of these laboratories consulted with SOWA-med (the Polish distributor of Labquality’s scheme) about the possible causes of deviating results and introduced changes in, for instance, the time and force of centrifugation, the manner of preparation of the control sample (very thorough mixing of the sample is required), the manner of sediment preparation, or the manner of calculation of results. Thus, it can be concluded that the EQA programme plays an important educational role in the implementation and monitoring of the standardisation of laboratory methods which is one of the main EQA scheme roles (*22*).

Our study has some limitations. Although we excluded from the analysis evident outliers which must have been caused by gross errors, we were not able to verify the impact on the quality of results in such factors as inappropriate control specimen preparation, inter-observer variability in particle recognition, different protocols of analyser results’ verification, cases of mixing of the WBC and RBC results or erroneous calculation of results. We did not analyse the performance for some method groups due to the unrepresentative, low number of results. We were not able to analyse the quality of results of manual methods according to the applied procedure (standardised and non-standardised), since a too small number of participants responded to the questionnaire. We did not perform the analysis without dividing the results according to the method group, because different target values have been reported for particular groups that could have been related to the different technical principles of methods (*17*). Centrifugation reduces the number of urine particle elements, and RBC and WBC results in coverslip or chamber counting from sediment methods can be lower in comparison with the direct counting method. Moreover, EQA specimens can be uncommutable and artefactual in some automated systems, since modified particles are used in control samples (*23*). For one of the analysers, in some surveys the applied technology did not permit it to recognise the particles in the control samples as well as in the other methods; however, this analyser was not evaluated due to the small number of results.

Despite these limitations, our analysis allows us to conclude that the process of implementation of the standardised manual urine particle counting procedures in the last ten years in Poland has not been sufficiently effective. The results of laboratories just starting to participate in the EQA scheme indicate that some laboratories still use traditional, non-quantitative methods. Our study should increase the awareness of low quality of urine particle counting results in laboratories, especially if manual methods are used, and will induce taking the actions aimed at improving the quality of results of this important laboratory test. In our opinion, the implementation of national guidelines for standardised urine particle counting, which is to be obligatory, seems very necessary and may result in a more effective implementation of standardised procedures that will affect the quality of urine particle counting results in all laboratories.
